# Upper tract urothelial carcinoma and bladder cancer in review in this number of International Brazilian Journal of Urology

**DOI:** 10.1590/S1677-5538.IBJU.2022.03.01

**Published:** 2022-04-14

**Affiliations:** 1 Universidade do Estado do Rio de Janeiro Unidade de Pesquisa Urogenital Rio de Janeiro RJ Brasil Unidade de Pesquisa Urogenital - Universidade do Estado do Rio de Janeiro - Uerj, Rio de Janeiro, RJ, Brasil; 2 Hospital Federal da Lagoa Serviço de Urologia Rio de Janeiro RJ Brasil Serviço de Urologia, Hospital Federal da Lagoa, Rio de Janeiro, RJ, Brasil

The May-June number of Int Braz J Urol, the 16th under my supervision, presents original contributions with a lot of interesting papers in different fields: Robotic Surgery, Prostate Cancer, Male Infertility, Overactive Bladder, Bladder Cancer, Upper urothelial carcinoma, renal stones, sexual dysfunction, PUV, Dysfunctional voiding and Uroanatomy. The papers came from many different countries such as Brazil, USA, Canada China, Italy, India, Denmark, Belgium and Egypt, and as usual the editor´s comment highlights some of them.

In the present issue we present two important reviews about upper urothelial carcinoma (UUC) and bladder cancer. The paper about UUC of the group of Dr. Sharma from India in page 406 shows a very complete systematic review about the topic (1). The authors shows that the tumor grade, stage, presence of lymphovascular invasion, lymph node metastasis, hydronephrosis, variant histology, sessile architecture, margin positivity and multifocality were associated with poor recurrence free survival (RFS), cancer-specific survival (CSS) and overall survival (OS). Presence of carcinoma in situ was associated with poor RFS and CSS but not OS. Tumor necrosis was associated with worst CSS and OS but not RFS. Tumor location was not a predictor of any of the survival parameters. Dr. Korkes and colleagues from Brazil and USA in page 397 (2) in a interesting narrative review about bladder cancer shows that patients with low-grade-non-muscle-invasive bladder cancer that TURBTs, chemoablation, BCG immunoablation, partial cystectomy, radical cystectomy, radiotherapy, and chemotherapy are attractive modalities to treat them effectively and proposes an algorithm to overcome these challenges. The editor in chief would like to highlight the following works too:

Dr. Mazzucchi and colleagues from Brazil and Canada, presented in page 456 (3) a nice review about the single use flexible ureteroscopes and concluded that these ureteroscopes are lighter and have superior quality of image when compared to fiberoptic ones and that there are no definite data showing a higher stone-free rate or less complications with the use of single-use flexible ureteroscopes.

Dr. Sobrinho and colleagues from the Urogenital Research Unit from Brazil performed in page 561 (4) a interesting translational study about the lower pole anatomy in anomalous kidneys and concluded that the knowledge of spatial anatomy of lower pole is of utmost importance during endourologic procedures in patients with kidney anomalies. The horseshoe kidneys had more restrictive anatomic factors in lower pole than the complete ureteral duplication.

Dr. Laursen and colleagues from Denmark and Brazil performed in page 471 (5) a nice study about the recombinant gonadotropin therapy to improve spermatogenesis in nonobstructive azoospermic patients and concluded that hormonal therapy with recombinant gonadotropins could be considered in infertile men with nonobstructive azoospermy as an alternative to sperm donation. Large-scale studies are needed to substantiate hormone stimulation therapy with recombinant gonadotropins in routine clinical practice for this severe form of male infertility.

Dr. Abdelhalim and colleagues from Egypt performed in page 485 the paper that is the cover in this edition (6). In this paper the authors assess the effect of bladder neck morphology and its incision (BNI) in patients with posterior urethral valve (PUV) on early reintervention rate and concluded that in morphologically high bladder neck associated PUV, concomitant BNI with posterior valve ablation doesn’t reduce early re-intervention rate.

Dr. Schulze and colleagues from Brazil performed in page 493 an important report about robotic surgery (7). The authors evaluated whether criteria exist to guide election between the use the three- or four-arm technique in robotic partial nephrectomy (RPN) instead of just the surgeon’s preference and concluded that the two robotic partial nephrectomy techniques had similar oncological and postoperative outcomes, with minimal perioperative complications. The three-arm technique is safe and feasible regardless of the complexity and size of the tumor. Additionally, the use of the three-arm technique reduced surgery costs by US$ 413.00 per patient.

The Editor-in-chief expects everyone to enjoy reading.
